# The role of zoonotic chlamydial agents in ruminants abortion

**Published:** 2017-10

**Authors:** Sara Barati, Naghmeh Moori-Bakhtiari, Masoud Ghorbanpoor Najafabadi, Hassan Momtaz, Leili Shokuhizadeh

**Affiliations:** 1 Department of Pathobiology, School of Veterinary Medicine, Shahid Chamran University of Ahvaz, Ahvaz, Iran; 2 Department of Pathobiology, School of Veterinary Medicine, Islamic Azad University, Shahrekord Branch, Sharekord, Iran; 3 Department of Microbiology, School of Medicine, Hamadan University of Medical Sciences, Hamadan, Iran

**Keywords:** Abortion, *Chlamydia*, Goat, PCR, Sheep

## Abstract

**Background and Objectives:**

Enzootic abortion of ewes (EAE) is caused by infection of sheep and goats by *Chlamydia abortus* bacterium. Chlamydial abortion in bovine could occur by *Chlamydia abortus, Chlamydia psittaci* and *Chlamydia pecorum. C. psittaci* is the causative agent of psittacosis or ornithosis disease in humans and birds. It also causes acute pneumonia in cattle and sheep. The present study aimed at surveying the role of chlamydial agents in ruminants abortion.

**Materials and Methods:**

A total of 117 aborted material samples (Cotyledon, liver, spleen, and abomasal contents of fetus) from 9 cattle and 100 sheep in Shahr-e-Kord and 8 sheep from Bagh-e-Malek were collected from different herds with abortion history during the lambing periods from 2014 to 2016. After DNA extraction, the samples were tested by species-specific PCR to detect *C. abortus, C. pecorum* and *C. psittaci.*

**Results:**

Out of 117 clinical sample (108 sheep and 9 cattle), chlamydial infection was detected in 66 (56.41%) samples by *Chlamydiales* order-specific primers. A total of 24 (36.36%) and 24 (36.36%) samples indicated positive forms of *C. abortus* and *C. psittasi* infections, respectively. Only 1 (1.5%) *C. pecorum* was identified from cattle using nested PCR during this study. Among 66 *Chlamydiales*-positive samples, 20 (30.30%) samples with coinfection of *C. abortus* and *C. psittaci* were detected, however, infection of 3 species was not detected in the samples.

**Conclusion:**

Because of the high percentage of chlamydial infection in these regions and probability of coinfection, conducting epidemiological studies on the role of different animals is highly recommended.

## INTRODUCTION

The family *Chlamydiaceae* contains obligate intra-cellular Gram-negative bacteria, with 11 confirmed species (*C. trachomatis, C. suis, C. psittaci, C. pneumoniae, C. pecorum, C. muridarum, C. gallinacea, C. felis, C. caviae, C. avium* and *C. abortus*) and candidate species (*C. ibidis*) relating to single genus of *Chlamydia* (1–4).

*C. abortus* is associated with enzootic abortion in ewes (EAE) (5). This is the most common infectious reason for abortion and the birth of weak lambs in many sheep-rearing countries of the world. Abortion usually occurs in the last 2 to 3 weeks of pregnancy. Animals that have been infected before pregnancy show no clinical signs of infection, with the organism arriving into a dormant phase. No clinical signs could be observed in the animals until abortion or delivery of very weak lambs. It was found that the abortion percentage in affected flocks is low in the first year and then reaches 30% and 10% in the second and third years, respectively (5). Hidden infections continuing longer than 3 years have also been described (6). Development of Chlamydiae is highly dependent on nutrient supply and the metabolic status of the host cell (7). Although *C. pecorum* is frequently isolated from the digestive tract of ruminants with no clinical symptoms, it is a causative agent of fertility disorder, conjunctivitis, arthritis, mastitis, and pulmonary inflammation in sheep, goats and cattle (8). While *C. psittaci* can cause severe flu-like infections in humans, birds develop largely non-specific, and sometimes, fatal intestinal and respiratory symptoms (9). Moreover, the disease affects goats, and to a lesser degree, cattle, horses, pigs and deer, while little is known about the rate of these infections because of lack of epidemiological evidences (10). Although *C. pecorum* association in small ruminants abortion incidents was formerly described nearly 20 years ago in south of France (11), its role as an etiological agent of abortion is not well-known in humans. *C. psittaci* comprises a range of *Chlamydia* with diverse genetic, serological, and host-tropic properties. By DNA-DNA hybridization examination, 14% to 95% homology was reported among *C. psittaci* strains (11) and less than 70% among mammalian strains, and avian strains of *C. psittaci.* Moreover, *C. abortus* strains are widespread among ruminants and have been related to abortion in horses, rabbits, guinea pigs, mice, pigs and humans (12).

In addition to DNA-based techniques (polymerase chain reaction and DNA microarray) and RFLP, various diagnosis techniques, such as direct microscopic inspection, culture in embryonated chicken eggs, or in cell cultures, serological exams for protein detection (complement fixation test (CFT), enzyme-linked immunosorbent assay (ELISA) and immunohisto-chemistry and direct immunofluorescence) could be utilized to recognize *Chlamydia* and *Chlamydia* in biological samples (13). Conventional and real-time PCR methods have been implemented using PCR, which amplify conserved regions of the chlamydial outer membrane protein genes *omp*A, *omp*1, and *omp*2, the polymorphic membrane gene *pmp*, genes, or the intergenic space between the 16S and 23S rRNA genes (14, 15). Several studies on *C. abortus* in sheep and goats by serology (16) and *C. psittaci* in pigeons (17) by PCR have been documented in Khuzestan province. Considering suspected *Chlamydia* abortion (last 2–3 weeks of pregnancy) in ruminants (bovine, ovine and goat) in the 2 mentioned provinces and migration of animals to and from these 2 provinces, the aim of this research was the primary study on the presence of important *Chlamydia* spp. in aborted ruminants with doubtful signs of *Chlamydia* abortion.

## MATERIALS AND METHODS

### Preparation of clinical samples.

A total of 117 aborted fetuses were collected from different herds located in southwest of Iran, where abortion had been observed during the lambing periods from 2014 to 2016. A total of 9 cattle and 100 sheep from Saman and Lordegan in Cheharmahal and Bakhtiari province and 8 sheep from Bagh-e-Malek in Khuzestan province were selected. Sampling was targeted, meaning that only aborted fetuses at the last 2 to 3 weeks of gestation were selected and transferred to the laboratory on ice. Sampling was performed in sterile conditions from liver, spleen, and abomasal contents of aborted fetus. Laborious methods were performed to ensure that tissues were collected from the same anatomical location in each animal. Strict aseptic protocols, including the use of new sets of tools, were used to avoid cross-contamination. The samples were stored in sterile microtubes at −20°C till DNA extraction.

### DNA extraction.

Genomic DNA was extracted from the tissue samples using a SinaGen Kit (Sina-Gen, Iran), according to the manufacturer’s instructions. Tissue samples were finely chopped using sterile blades prior to extracting DNA. Genomic DNA extracted from each isolate was quantified using the Nano Drop spectrophotometer and stored in −20°C for the next genomic evaluation.

### PCR assay.

Precautions were taken to use sterile reagents and conditions; and contamination of reactions by PCR product was avoided by strict separation of working areas. The optimal PCR conditions for *C. abortus, C. psittaci* and *C. pecorum* individual amplification were initially determined separately using serial dilutions of respective DNA solution. The PCR reactions were performed in a final volume of 25 μL containing 12.5 microliter of master mix 2× (Ampliquen, Denmark) containing 1× PCR buffer, 200 μM of 4 deoxynucleoside triphosphate (dNTPs), 2 mM MgCl_2_, and 0.5 U of *Taq* polymerase, then, 0.5 μM of each primer set and 2 microliter of extracted DNA were added to each reaction. PCR reactions were performed in an Eppendorf thermocycler (Eppendorf, Germany). Thermal conditions for amplification of *Chlamydiales* specific gene were initial denaturation for 5 minutes at 95°C, 39 one-minute cycles at 94°C, 45 seconds at an annealing temperature of 54°C, and elongation for 45 seconds at 72°C, with a final extension step at 72°C for 5 minutes. The PCR products were subjected to electrophoresis for 1 hour at 70V in 1.5% safe stain containing aga-rose gel, and the results were visualized and photographed under ultraviolet illumination. Detection of *C. pecorum* infection of samples was conducted by Nested-PCR. The name, sequence and the predicted amplified fragment of studied genes, as well as the annealing temperature are listed in Table 1. The standard strain DNA of *C. abortus* S26/3 and *C. pecorum* W73, obtained from Professor Borel (University of Zurich) as a gift, and *C. psittaci* 6BC, as obtained from Professor Sarryopoglu (University of Turkey) as gift, were used as positive controls for each round of PCR (18–20).

**Table 1.** Primers Used to Detect *Chlamydia* Bacterium in Aborted Fetus

**Gene**	**Sequences**	**Segment (bp)**	**Ref.**
*Chlamydiales* (16s–23s spacer region)	F: 5-CAAGGTGAGGCTGATGAC-3	352	(18)
	R: 5-TCGCCTKTCAATGCCAAG-3		
*C. abortus* (16srRNA)	F: 5′-TGG TAT TCTTGC CGA TGA C-3′	479	(19)
	R: 5′-GAT CGT AAC TGC TTA ATA AAC CG-3′		
*C. psittaci* (*pmp* gene)	F: 5′-ATG AAA CAT CCA GTC TAC TGG-3′	300	(13)
	R: 5′-TTG TGT AGT AAT ATT ATC AAA-3′		
*C. pecorum (momp)*	F: 5-GCICTITGGGAATGCGGITGCGCIAC-3	576–597	(20)
	R: 5-TTAGAAICGGAATTGIGCATTIACGTGIGCICG-3		
	F: 5-CCAATACGCACAATCGAAACCTCGC-3	426–441	
	R:5-CCACAAAATTTTCTAGACTTCAACTTGTTAAT-3		

## RESULTS

**Fig 1.**
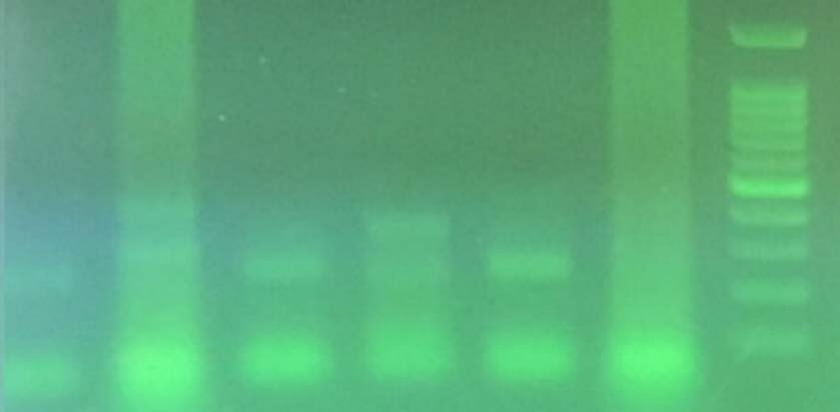
Result of PCR using *C. psittaci* specific primers: right to left: 100 bp DNA ladder; negative control; positive control (300 bp); 4 samples

**Fig. 3.**
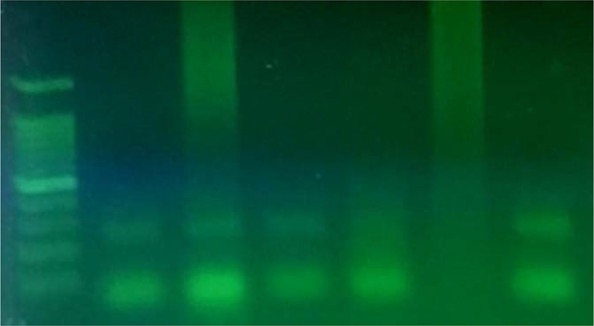
Agarose gel electrophoresis of *C. abortus* S26/3 species-specific conventional PCR: left to right: 100 bp DNA ladder; 3 positive samples; 1 negative sample; negative control; positive control (222bp).

The samples were tested by conventional PCR to identify specific *16S rRNA* and *pmp* genes of *C. abortus* and *C. psittaci*, respectively. As expected, PCR amplification of DNA for *C. abortus* produced 222bp fragment and produced 300 bp fragments for *C. psittaci*. The annealing temperature of 54°C and 48°C were used for these PCR experiments, respectively (Figs. 1, 3).

**Fig. 2.**
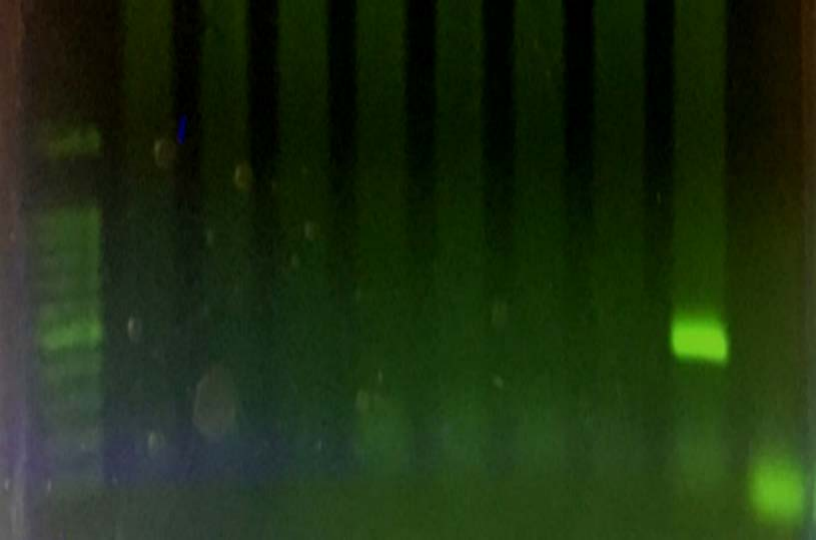
Agarose gel electrophoresis of *C. pecorum* species-specific Nested-PCR: left to right: 100 bp DNA ladder; seven negative samples; positive control (426–441 bp); negative control (distilled water).

Out of 117 doubtful chlamydial clinical samples taken from the infected animals (108 sheep and 9 cow), 66 (56.41 %) samples were detected by either one of the 3 pathogens. A total of 24 (36.36%) and 24 (36.36%) sheep samples were positive for *C. abortus* and *C. psittasi*, respectively. In this study, only 1 (1.5%) *C. pecorum* was identified from cattle by producing a 576–597 and 426–441 bp fragment using Nested-PCR. Annealing temperatures used in the first and second stages were 52°C and 50°C, respectively (Fig. 2).

The specificity of the PCR experiments using these primers were checked on genomic DNA samples from unrelated bacteria. None of the DNA samples from non-chlamydial bacteria created a measurable PCR bands in these experiments. No PCR product was produced using water instead of target DNA. The results are demonstrated in Table 2.

**Table 2.** Results of Chlamydial Infection of the Studied Samples

**Total of samples**	**Positive number of *Chlamydiales* order**	***Cp. abortus***	***Cp. psittasi***	***Cp. pecorum***	***Coinfection (Cl. abortus+Cl. psittasi)***	***Coinfection (Cl. abortus+Cl. psittasi + Cl. pecorum)***
117	66	24	24	1	20	0
(9 cattle +108 sheep)	(56.41%)	(36.36%)	(36.36%)	(1.5%)	(30.30%)	(0%)

## DISCUSSION

*Chlamydiaceae* family is considered as one of the main bacterium related to abortion in ruminants, such as sheep, goats, and cattle (21). Abortion is economically important in many herds of sheep and goats in Europe, North America, Africa, and Iran. The bacteria causes premature birth, reproductive disorders in ruminants, inflammation of the epididymis, pneumonia, arthritis, and conjunctivitis in the feces of healthy sheep and goats (22); also, it is a zoonotic risk for numerous pregnant women. It is reported that *C. abortus* can be spread in human placenta (9). This bacterial family is remarkably important. Thus, many studies have been conducted to identify and recognize these bacteria. For example, the prevalence of infection with this bacterium was reported to be 8.9% in a serological study by ELISA in sheep of Ahvaz, Iran (16). In house ELISA kit, based on rPOMP-90-3, 4 and 3+4 antigens were designed by Bakhtiari et al. to prevent available cross-reaction between *C. abortus* and *C. pecorum* in commercial kits (23). Moreover, in Mahzouniyeh et al. research (2014), *C. abortus* contamination in Shahr-e-Kord was reported to be 52% using Nested-PCR (24). In 2009, Pantchev et al. detected *C. psittaci* and *C. abortus* based on *omp*A gene from tissue samples using real-time PCR (25). Regular methods, such as bacterial culturing and staining, are slightly more sensitive in detecting *Chlamydia* bacterium in field samples. However, these methods are uncertain in most situations and are more difficult. The new development of different PCR assays has been described to detect *Chlamydia* bacterium in samples from the aborted fetuses (26). PCR provides a rapid diagnosis without the need for a culture or identifying species and strains with more similarity. Also, PCR detection is not affected by the lack of viability of the microorganism and is more sensitive than culture in detecting nonfeasible organisms and cellular DNA. Previous results have revealed that the PCR amplification of *16S rRNA* genes is a good target for identifying *Chlamydia* spp. (15). Although there are different sets of primers that allow the identification of all species of the *Chlamydiaceae* family, PCR assays that amplify segments of the *16S rRNA* genes present high sensitivity and specificity (27). Based on present results, *C. abortus, C. psittaci* and *C. pecorum* can be differentiated by PCR products obtained with species-specific primers to *16S rRNA, pmp* and *momp* gene. The specificity of those primers allows the differentiation of *C. abortus* and *C. pecorum* using a conventional PCR. The fact that a considerable proportion of sheep samples (20 of 57 positive samples) were contaminated with 2 chlamydial agents is in line with previous study. The clinical features of abortion caused by *C. abortus* and *C. psittaci* are highly similar and such mixed infections have been proposed to be a common incidence in sheep and goat herds (28). Investigation of a large panel of diagnostic samples revealed an interesting epidemiological aspect, which was the occurrence of 2 chlamydial species in 1 sample. This was in agreement with previous findings (29) that reported the same species in pigs suffering from respiratory symptoms or fertility problems. Moreover, infections caused by *C. suis, C. abortus, C. pecorum* and *C. psittaci* were reported (30, 31). The existing data suggest that the sheep seem to be a host mainly susceptible to co-infections. In the present study, combinations of *C. abortus* and *C. psittasi* (35.08%) were regularly identified in sheep samples. A certain preference of *C. abortus* and *C. psittasi* to perform in concert with another chlamydial agent has already been reported (32). How does a bacterium that causes systemic disease in birds transform into an organism of mammalian abortion? The response will offer important visions into the mechanisms of chlamydial virulence and can finally be answered by genome sequence comparison. Until then, our capability to differentiate *C. psittaci* and *C. abortus* will remain to rely on ecological alterations, mAbs, and genetic data (16S or 23S rRNA signature sequences), and *omp*A, cysteine-rich proteins (27, 33, 34). In this study, PCR-amplification of *momp* gene, using specie-specific primers by nested-PCR, identified *C. pecorum* strain in cattle. Another study revealed that *C. pecorum* was more widespread in cattle than *C. abortus* and that the bacteria were frequently detected in vaginal swabs and fecal samples (35). Earlier data on *C. pecorum* involvement in abortion in Tunisia and Morocco indicated that *C. pecorum* may cause abortion in small ruminants in North African countries. Several studies have indicated that *C. pecorum* can also be a possible reason of abortion in ewes and goats (36). Clinically unclear intestinal infections produced by *C. pecorum* have already been reported in both abortion-affected and unaffected ruminant flocks (37). Also, the mixed infection of *C. pecorum* with *C. abortus* related to abortion in water buffalo in south of Italy (38) suggests that *C. pecorum* could also be associated with abortion in large ruminants. Consequently, differentiating the 2 species in abortion material is highly necessary. Nevertheless, it is still unknown whether *C. pecorum*-related abortion is a consequence of *C. pecorum* alone or is due to a development of its pathogenesis mediated by the coinfection with *C. abortus*; its pathogenicity may be related to a lack of nutrients or parasitic invasions, which frequently occur in these countries. It could also be considered that no pathogenic *C. pecorum* strains might be spread from the intestine through the blood circulation and reach the placenta, where they cause abortion due to some unidentified physiopathologic situations. The presence of 1 *C. pecorum* among 66 samples included in our study suggests that abortion by *C. pecorum* is rare in the region. Also, migration of the flocks toward Baghe-Malek and Shahr-e-Kord during winter and summer can cause co-contamination and simultaneous infection in these areas. Thus, as co-infections are not rare events, the combination of various specific diagnostic tests is crucial for epidemiological studies.

## References

[1] Kuo CC Stephens RS Bavoil PM Kaltenboeck B Genus I. Chlamydia. In: Krieg N Staley J Brown D Hedlund B Paster B Ward W editors. Bergey’s Manual of Systematic Bacteriology. 2nd ed vol 4. New York, USA: Springer-Verlag; 2011 pp. 846–865.

[2] Sachse K Laroucau K Riege K Wehner S Dilcher M Creasy HH Evidence for the existence of two new members of the family *Chlamydiaceae* and proposal of *Chlamydia avium* sp. nov. and *Chlamydia gallinacea* sp. nov. Syst Appl Microbiol 2014; 37:79–88. 24461712 10.1016/j.syapm.2013.12.004

[3] Vorimore F Hsia RC Huot-Creasy H Bastian S Deruyter L Passet A Isolation of a new *Chlamydia* species from the feral Sacred Ibis (Threskiornis aethiopicus): *Chlamydia ibidis*. PLoS One 2013; 8(9): e74823. 24073223 10.1371/journal.pone.0074823 PMC3779242

[4] Siarkou VI Vorimore F Vicari N Magnino S Rodolakis A Pannekoek Y Diversification and distribution of ruminant *Chlamydia abortus* clones assessed by MLST and MLVA. PLoS One 2015; 10: e0126433. 26001070 10.1371/journal.pone.0126433 PMC4441495

[5] Victoria S Alexandros FL Sofia Ch Kotsis A Papadopoulos O Subspecies variation in Greek strains of *Chlamydophila abortus*. Vet Microbiol 2002; 85: 145–157. 11844621 10.1016/s0378-1135(01)00506-5

[6] Schiller I Koesters R Weilenmann R Thoma R Kaltenboeck B Heitz P Mixed infections with porcine *Chlamydia trachomatis/pecorum* and infections with ruminant *Chlamydia psittaci* serovar 1 associated with abortions in swine. Vet Microbiol 1997; 58: 251–260. 9453135 10.1016/s0378-1135(97)00154-5

[7] Wang C Gao D Kaltenboeck B Acute *Chlamydia pneumonia* re-infection accelerates the development of insulin resistance and diabetes in obese C57BL6 mice. J Infect Dis 2009; 200: 279–287. 19508161 10.1086/599796

[8] Reinhold P Jaeger J Liebler-Teneorio E Berndt A Bachmann R Schubert E Imapct of latent infections with *Chlamydophila* species in young cattle. Vet J 2008; 175: 202–211. 17317243 10.1016/j.tvjl.2007.01.004

[9] Buxton D Anderson IE Longbottom D Livingstone M Wattegedera S Entrican G Ovine chlamydial abortion: characterization of the inflammatory immune response in placental tissues. J Comp Pathol 2002;127:133–141. 12354524 10.1053/jcpa.2002.0573

[10] Longbottom D Coulter LJ Animal *Chlamydioses* and zoonotic implications. J Comp Pathol 2003;128:217–244. 12834606 10.1053/jcpa.2002.0629

[11] Rodolakis A Salinas J Papp J Recent advances on ovine chlamydial abortion. Vet Res 1998; 29: 275–288. 9689742

[12] Fukushi H Hirai K Genetic diversity of avian and mammalian *Chlamydia psittaci* strains and relation to host origin. J Bacteriol 1989; 171: 2850–2855. 2565333 10.1128/jb.171.5.2850-2855.1989 PMC209973

[13] Laroucau K Trichereau A Vorimore F Mahé AM A pmp genes based PCR as a valuable tool for the diagnosis of avian *Chlamydiosis*. Vet Microbiol 2007; 121: 150–157. 17169505 10.1016/j.vetmic.2006.11.013

[14] Everett KD Chlamydial and Chlamydiales: more than meet the eye. Vet Microbiol 2000; 75: 109–126. 10889402 10.1016/s0378-1135(00)00213-3

[15] Madico G Quinn TC Boman J Gaydos CA Touch-down enzyme time release-PCR for detection and identification of *Chlamydia trachomatis, Chlamydia pneumoniae* and *Chlamydia psittaci*: using the 16S-23S spacer rRNA genes. J Clin Microbiol 2000;38:1085–1093. 10699002 10.1128/jcm.38.3.1085-1093.2000 PMC86346

[16] Ghorbanpoor M Goraninejad D Heydari R Serological study on enzootic abortion of ewes in Ahvaz, Iran. Anim Vet Adv 2007; 6: 1194–1196.

[17] Ghorbanpoor M Moori Bakhtiari N Mayahi M Hana Moridveisi Detection of *Chlamydophila psittaci* from pigeons by polymerase chain reaction in Ahvaz. Iran J Microbiol 2015; 7: 18–22. 26644869 PMC4670462

[18] Sachse K Laroucau K Vorimore F Magnino S Feige J Müller W DNA microarray-based genotyping of *Chlamydophila psittaci* strains from culture and clinical samples. Vet Microbiol 2009; 135: 22–30. 18950965 10.1016/j.vetmic.2008.09.041

[19] Longbottom D Fairley S Chapman T Psarrou E Vretou E Livingstone M Serological diagnosis of ovine enzootic abortion by enzyme-linked immunosorbent assay with a recombinant protein fragment of the Polymorphic outer membrane protein POMP90 of *Chlamydophila abortus*. J Clin Microbiol 2002; 40(11): 4235–4243. 12409404 10.1128/JCM.40.11.4235-4243.2002 PMC139646

[20] Sachse K Hotzel H Detection and differentiation of *Chlamydiae* by Nested PCR. Detection of microbial pathogens. Methods Mol Biol 2003; 216:123–136. 12512360 10.1385/1-59259-344-5:123

[21] DeGraves FJ Gao D Hehnen HR Schlapp T Kaltenboeck B Quantitative Detection of *Chlamydia psittaci* and *C. pecorum* by high-sensitivity real-time PCR reveals high prevalence of vaginal infection in Cattle. J Clin Microbiol 2003; 41: 1726–1729. 12682170 10.1128/JCM.41.4.1726-1729.2003 PMC153858

[22] Creelan JL McCullough SJ Evaluation of strain-speciec primer sequences from an abortifacient strain of ovine *Chlamydophila abortus (Chlamydia psittaci)* for the detection of EAE by PCR. FEMS Microbiol Lett 2000; 190(1):103–108. 10981698 10.1111/j.1574-6968.2000.tb09270.x

[23] Moori Bakhtiari N Seifi M Ghorbanpour M Gooraninejad S Cloning and expression segment of the POMP90 gene *Chlamydophila abortus* strain S26? 3 in *E. coli*. Iran Vet J 2011; 7: 74–80.

[24] Mahzouniyeh M Golboui daghdari SH Pourahmad R Detection of *Chlamydophila abortus* abortions in sheep in the Chaharmahal-va-Bakhtiyari province, using Nested PCR. Vet J 2014; 2: 80–74.

[25] Pantchev A Sting R Bauerfeind R New real-time PCR tests for species-specific detection of *Chlamydophila psittaci* and *Chlamydophila abortus* from tissue samples. Vet J 2009; 181: 145–150. 18413292 10.1016/j.tvjl.2008.02.025

[26] Pelletier C Chartier S Berthillier J Spohr H Carvalho Lima BAD Negrão FJ Validation of an internal method for the diagnosis of infections with *Chlamydophila abortus* and *Coxiella burnetii* by real-time multiplex PCR. Dev Biol (Basel) 2006; 126: 219–226. 17058498

[27] Meijer A Kwakke GJ de Vries A Schouls LM Ossewaarde JM Species identification of *Chlamydia* isolates by analyzing restriction fragment length polymorphism of the 16S-23S rRNA spacer region. J Clin Microbiol 1997; 35:1179–1183. 9114403 10.1128/jcm.35.5.1179-1183.1997 PMC232725

[28] Aitken ID Clarkson MJ Linklater K Enzootic abortion of ewes. Vet Rec 1990; 126: 136–138. 2316135 10.1136/vr.126.6.136

[29] Hoelzle LE Steinhausen G Wittenbrink MM PCR-based detection of chlamydial infection in swine and subsequent PCR-coupled genotyping of chlamydial ompA-Gene amplicons by DNA-hybridization, RFLP analysis, and nucleotide sequence analysis. Epidemiol Infect 2000; 125: 427–439. 11117968 10.1017/s0950268899004446 PMC2869617

[30] Teankum K Pospischil A Janett F Burgi E Brugnera E Hoelzle K Detection of chlamydiae in boar semen and genital tracts. Vet Microbiol 2006; 116(1–3):149–157. 16650659 10.1016/j.vetmic.2006.03.021

[31] Kauffold J Melzer F Henning K Schulze K Leiding C Sachse K Prevalence of chlamydiae in boars and semen used for artificial insemination. Theriogenology 2006; 65: 1750–1758. 16280160 10.1016/j.theriogenology.2005.10.010

[32] Pantchev A Sting R Bauerfeind R Tyczka J Sachse K Detection of all *Chlamydophila* and *Chlamydia* spp. of veterinary interest using species-specific real-time PCR assays. Comp Immunol Microbiol Infect Dis 2010; 33: 473–484. 19733907 10.1016/j.cimid.2009.08.002

[33] Herrmann B Pettersson B Everett KD Mikkelsen NE Kirsebom LA Characterization of the rnpB gene and the RNase P RNA in the order *Chlamydiales*. Int J Syst Evol Microbiol 2000; 50 Pt 1:149–158. 10826799 10.1099/00207713-50-1-149

[34] Bush RM Everett KDE Molecular evolution of the *Chlamydiaceae*. Int J Syst Evol Microbiol 2001; 51(Pt 1):203–220. 11211261 10.1099/00207713-51-1-203

[35] Berri M Rekiki A Boumedine KS Rodolakis A Simultaneous differential detection of *Chlamydophila abortus, Chlamydophila pecorum* and *Coxiella burentii* from aborted ruminant’s clinical samples using Multiplex PCR. BMC Microbiol 2009; 9: 130–138. 19570194 10.1186/1471-2180-9-130 PMC2725139

[36] Rekiki A Bouakane A Hammami S El Idrissi AH Bernard F Rodolakis A Efficacy of live *Chlamydophila abortus vaccine* 1B in protecting mice placentas and foetuses against strains of *Chlamydophila pecorum* isolated from cases of abortion. Vet Microbiol 2004; 99:295–299. 15066732 10.1016/j.vetmic.2004.01.010

[37] Kaltenboeck B Hehnen HR Vaglenov A Bovine *Chlamydophila* spp. Infection: Do we underestimate the impact on fertility? Vet Res Commun 2005; 29 Suppl 1:1–15. 10.1007/s11259-005-0832-4 PMC7088619 15943061

[38] Greco G Corrente M Buonavoglia D Campanile G Di Palo R Martella V Epizootic abortion related to infections by *Chlamydophila abortus* and *Chlamydophila pecorum* in water buffalo (Bubalus bubalis). Theriogenology 2008; 69: 1061–1069. 18374406 10.1016/j.theriogenology.2008.01.018

